# Improving social accountability processes in the health sector in sub-Saharan Africa: a systematic review

**DOI:** 10.1186/s12889-018-5407-8

**Published:** 2018-04-13

**Authors:** Georges Danhoundo, Khalidha Nasiri, Mary E. Wiktorowicz

**Affiliations:** 1 Faculty of Health (York University), 435 Health, Nursing & Environmental Studies Bldg, 4700 Keele St., Toronto, ON M3J 1P3 Canada; 2 Dahdaleh Institute for Global Health Research, Community and Global Health, Health Policy and Management, Faculty of Health, York University, Toronto, Canada

**Keywords:** Social accountability, Enabling factors, Limiting factors, Community, Citizen engagement, Sub-Saharan Africa

## Abstract

**Background:**

Social accountability is a participatory process in which citizens are engaged to hold politicians, policy makers and public officials accountable for the services that they provide. In the Fifteenth Ordinary Session of the Assembly of the African Union, African leaders recognized the need for strong, decentralized health programs with linkages to civil society and private sector entities, full community participation in program design and implementation, and adaptive approaches to local political, socio-cultural and administrative environments. Despite the increasing use of social accountability, there is limited evidence on how it has been used in the health sector. The objective of this systematic review was to identify the conditions that facilitate effective social accountability in sub-Saharan Africa.

**Methods:**

Electronic databases (MEDLINE, PsycINFO, Sociological Abstracts, Social Sciences Abstracts) were searched for relevant articles published between 2000 and August 2017. Studies were eligible for inclusion if they were peer-reviewed English language publications describing a social accountability intervention in sub-Saharan Africa. Qualitative and quantitative study designs were eligible.

**Results:**

Fourteen relevant studies were included in the review. The findings indicate that effective social accountability interventions involve leveraging partnerships and building coalitions; being context-appropriate; integrating data and information collection and analysis; clearly defined roles, standards, and responsibilities of leaders; and meaningful citizen engagement. Health system barriers, corruption, fear of reprisal, and limited funding appear to be major challenges to effective social accountability interventions.

**Conclusion:**

Although global accountability standards play an important guiding role, the successful implementation of global health initiatives depend on national contexts.

**Electronic supplementary material:**

The online version of this article (10.1186/s12889-018-5407-8) contains supplementary material, which is available to authorized users.

## Background

In sub-Saharan Africa, concerns have been raised regarding the quality of services delivered and health outcomes [1]. Existing health system bottlenecks such as drug shortages [2, 3], disrespect of patients in public health facilities [4], health workers’ focus on donor-funded activities that offer access to per diems [5, 6], and drug and bed net pilfering [7, 8] are among the factors that affect health service functioning in sub-Saharan African countries. The 2008 Accra Agenda for Action and the 2005 Paris Declaration on aid effectiveness emphasized country ownership for development policies through citizen engagement. Social accountability is a process in which citizens are engaged to hold politicians, policy makers public officials accountable for the services that they provide. It can be defined as “an approach towards building accountability that relies on civic engagement, i.e., in which it is ordinary citizens and/or civil society organizations who participate directly or indirectly in exacting accountability” [9]. In the context of health care, social accountability is a form of participatory citizen engagement in which citizens are recognized as service users who are ultimately impacted by health care decisions and thereby can affect change in health policies, health services and/or health provider behaviour through their collective influence and action [10]. Scholars consider two key aspects of social accountability: answerability and enforceability [11, 12]. Answerability is the obligation of politicians, policy makers, and providers to explain and justify their actions. This includes being answerable for meeting performance objectives, measured against a number of goals or standards in a complex relationship that involves several stakeholders with vested interests and different levels of authority [13]. Enforceability refers to the capacity to ensure an action is taken and can involve penalties, consequences or remedies for failure to do so*.* In many sub-Saharan African countries, community participation, especially women’s, in accountability processes is fragmented [2, 14, 15]. Social accountability can play an important role in addressing corruption, increasing trust in public servants and government, which is key to accelerating efforts to achieve the Sustainable Development Goals (SDGs), and increasing the power and influence of citizens on agenda-setting [16–19]. Identifying the conditions for implementing successful social accountability initiatives can help community leaders, civil society organizations (CSOs), or non-governmental organizations (NGOs), to increase their leverage. While there have been several studies examining social accountability initiatives on health outcomes in various sub-Saharan African countries, there has been no systematic analysis of these initiatives in aggregate to identify common enabling and limiting factors to success. The objective of this paper is to analyze the conditions that foster effective social accountability initiatives in sub-Saharan African countries.

## Methods

### Search strategy and selection criteria

We systematically reviewed the published literature from 2000 to 2017 to identify studies regarding social accountability interventions in sub-Saharan Africa. Literature searches were carried out in the electronic databases Ovid MEDLINE, Social Sciences Abstracts, Sociological Abstracts, and PsycINFO. Search terms included combinations of: social accountability, accountability, citizen-led accountability, citizen voice, health sector, community participation, community engagement, sub-Saharan Africa, and Africa. The search terms were used in combination with the Boolean operators AND, OR, and * (asterisks).

The PRISMA criteria for reporting systematic reviews was followed [20]. We initially screened all unique publications for eligibility based on relevance of title and abstract. We included quantitative (e.g., randomized controlled trials) and qualitative studies (e.g., observational and ethnographic studies) published in the English language in which the population of interest was in sub-Saharan Africa. Studies that examined a social accountability intervention exclusively or as part of a larger study were included. Studies in which the accountability intervention did not involve a significant citizen engagement component were excluded. Other exclusion criteria included documents published in a non-English language, grey literature, theses, reports, protocols, proposals, editorials and non-peer-reviewed publications.

### Data abstraction

Data were recorded on country and location of intervention, social accountability intervention, participant characteristics, facilitator characteristics, study design, intervention characteristics, study outcome measures, reported results, and enabling and limiting factors identified. Study quality was assessed using a checklist and included assessment of use of a control group and randomization (for intervention studies), objectivity of outcome measures, adequate method of analysis described (for qualitative studies), and description of the population of interest.

## Results

**Fig. 1:**
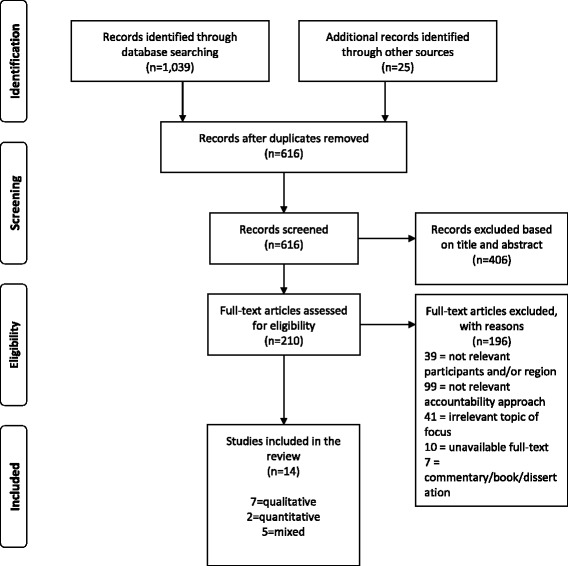
Search, screening, selection, and inclusion process diagram

The screening and selection process is outlined in Fig. 1. The initial search identified 1039 potentially relevant papers. Twenty-five additional papers were identified by looking through the references of included studies; 448 papers were removed due to duplication, leaving 616 papers for screening. Based on title and abstract, 406 papers were discounted as not relevant based on the inclusion criteria. Of the remaining 210 papers for which the full-text was reviewed, 196 were removed due to either irrelevant region or participants of focus (e.g., high income countries), irrelevant topic or accountability approach (i.e., looking at other forms of accountability other than social accountability), unavailable full-text, or being a commentary, book or dissertation. The remaining 14 studies met inclusion criteria and were included in the final review: 7 qualitative [14, 21–26], 5 mixed methods [27–31] and 2 quantitative articles [32, 33].

Additional file 1 summarizes the characteristics of the included studies. Study locations included Kenya [27, 30], Uganda [22, 32], Ghana [21, 22], Zambia [29], Tanzania [29], Benin [23], Guinea [23], the Democratic Republic of Congo (DRC; [23–26]), Malawi [33], Sierra Leone [14], and Nigeria [14, 31].

### Quality assessment of included studies

The methodological quality of the studies is summarized in Additional file 2. Study methodological quality ranged from moderate to very good. Three (21.4%) of studies used a controlled intervention design [30, 32, 33], and one (7.1%) used an uncontrolled intervention design [28]. Four (28.6%) studies did not include a baseline assessment [14, 21, 23, 27]. Two (50.0%) of four intervention studies did not include a control group [28, 32], and two (14.2%) studies did not clearly specify their outcome measures [14, 21].

### Approaches to social accountability

A variety of social accountability approaches were used. Eight studies (57.1%) used health facility committees as their primary social accountability mechanism [21, 23–26, 29–31], one (7.1%) used a health facility charter [27], two (14.2%) used citizen report cards [22, 32], and three (21.4%) used scorecards [14, 28, 33]. Two (14.2%) studies explored perspectives on a variety of social accountability mechanisms [24, 25].

### Enabling and limiting factors to social accountability initiatives

Study outcomes are reported in Additional file 1. Two (14.2%) studies used household surveys [27, 32], three (21.4%) used questionnaires to assess their health facility and care experiences [28, 31, 33], and one (7.1%) used a questionnaire to assess health indicators [30]. The remainder used qualitative analytic approaches such as content or thematic analysis to interpret focus group or interview discussions [22, 24–29, 31].

#### Successful interventions

Eleven (78.6%) of articles reported overall positive results from their social accountability approach.

##### Health committees

Of the eight articles using health committees as the social accountability approach, six reported overall success.

In Kenya, dialogue and engagement between the service delivery system and communities served via health facility committees in six districts proved to be a significant factor for improving certain health indicators [30]. After two years, intervention sites had significantly higher immunization coverage (91%) compared to control sites (66%). Several other health indicators improved more at intervention sites compared to control sites (i.e., health facility deliveries, insecticide-treated bed net use, latrine presence, food availability, and water treatment; see Additional file 1 for specific results). Breaking down the results by district, however, reveals that in four out of six districts, the intervention was unable to change the low rate of health facility birth deliveries at any of the sites. Further, there was no difference in family planning rates for both conditions, although this was not a statistically significant finding. Clear roles and functions of health committees, representativeness and inclusiveness of community in health committees, sustainability of improvements, and valid data sources were identified as facilitating factors. Poor capacity for data management and lack of community-targeted initiatives were identified as limiting factors.

In Nigeria, Uzochukwu et al. [31] found overall positive effects of both village-level and district-level health committees. For example, 89.3% and 100% of village- and district- level committee members, respectively, reporting observing changes in provision of drugs and 100% felt they should participate in community mobilization. Between 7.1 and 35.7% of community members reported not participating in health activities, such as community mobilization of health programs and identifying health needs in the community, because of religious differences, political issues, fear of government stopping funding, and opposition to committee leadership. The authors stressed the importance of involving citizens in the decision-making process early and also found that district and village health committees were dysfunctional in part due to a lack of understanding and recognition of their roles. Lodenstein et al. [23] compared 11 health facility committees across Guinea, Benin and DRC and noted various ways that they could facilitate social accountability: they initiate information and data collection, provide a forum for dialogue, ensure consequences and follow-up of complaints, and provide feedback to the community.

In the DRC, through interviews with 35 community members and health officials, Mafuta and colleagues [25] identified the following factors as facilitators of social accountability initiatives: community associations and groups, experiences in social mobilization and networking, cultural diversity and marginalized population, women’s status and participation in community groups’ activities, existing media and access to information, supportive regulatory environment, resources, and negotiation ability. They also identified certain contextual factors that limit social accountability initiatives: lack of networks, insufficient capacity for community mobilization, poor socioeconomic conditions (e.g. poor wages, lack of safe water and electricity), lack of radio and media coverage in rural levels, and poor negotiation ability. Mafuta et al. [26] made similar identifications, in addition to emphasizing the support of health zone management teams in community participation activities and improving the attitude of health providers towards voice at the health facility level. Mafuta et al. [24] showed the importance of considering contextual factors. For example, six out of 20 women from two health zones in the DRC cited it being customary for people not to complain as a cultural factor that prevented them from raising their concerns.

##### Scorecards

In Ghana, one year after the development of scorecards to improve maternal and newborn health services at 37 health facilities, a 41% increase in essential drugs rating, 22% increase in infrastructure rating, 47% increase in accessibility and access to information rating, 14% increase in water, sanitation, and hygiene rating, and an 18% increase in essential equipment rating were reported by citizens, community leaders, and health and non-health stakeholders [28]. The authors assessed whether engaging multiple health and non-health stakeholders resulted in improvements. They documented that engaging a broad range of stakeholders, including citizens, in social accountability initiatives targeting local health facilities can lead to improvements in maternal and newborn health services due to a heightened sense of shared ownership. They also identified higher levels of community engagement in districts where the chiefs of maternal and newborn health councils were engaged. The authors noted that successful implementation could be limited by lack of external financial and technical inputs, weak community leadership, and lack of sustainability of this intervention without continued commitment from community members to remain engaged and widen its reach.

In Nigeria, scorecards highlighting maternal, newborn, and child health indicators that were developed by state officials in collaboration with CSOs, media, community advocates, and health professionals were successfully used to increase the health budget from 8% in 2014 to 15% in 2016 [14]. The scorecards were promoted through social media campaigns and pressured electoral candidates. This case demonstrates the utility of strategic partnerships between multiple stakeholders at the national level. In Sierra Leone, the use of scorecards gave voters and politicians access to health financing evidence (including mismanagement). As a result, 5 out of 6 political parties signed a “Health Manifesto” and 68 parliamentarians signed pledge cards [14]. Leveraging social accountability tools such as the media has proved to facilitate social accountability by relaying information on the roles and responsibilities of various officials. These specific commitments by candidates were broadcast on television and radio to ensure citizens were aware of the specific responsibilities they agreed to take on once elected. The study authors note that significant time and data were required in order to successfully implement the mechanism.

In Malawi, in a two-year cluster-randomized control trial, Gullo et al. [33] found several improvements on scorecard indicators. In the intervention group, CHW visits to pregnant women increased by 20% and by 6% in the postnatal period, compared to the control group. There were also significant increases in ratings on the relationship between health provider and community (37%), availability and accessibility of reproductive and maternal health information (22%), commitment of service providers (26%), level of youth and male involvement (23 and 33%, respectively), availability of referral transportation (21%), and women’s satisfaction with health services. The authors document that facilitating factors for success included instilling communication, trust, responsiveness and quality of patient-provider interactions, as well as emphasizing locally-relevant solutions to improving access.

##### Citizen report cards

In addition to scorecards, citizen report cards (CRCs) have also been successfully used as a social accountability tool. Björkman and Svensson [32] conducted a randomized field experiment in nine Ugandan districts using citizen report cards as a social accountability intervention to elicit health provider and staff behaviour change. Report cards were developed that summarized the community’s perception of various issues. Results demonstrated significantly improved uptake of health services and health outcomes. For example, after one year, 36% of treatment facilities had suggestion boxes while no control facilities had them. Seventy percent of treatment facilities also posted information on free services and patients’ rights, while only 4 out of 25 control clinics did so. Furthermore, waiting times to see a health provider were 119 min in the treatment facilities compared to 131 min in control facilities. The absentee rate was 13 percentage points lower in the treatment facility. With regards to health service uptake, the authors reported significantly higher child immunization rates in the treatment group as compared to the control group and a 33% reduction in the under-five mortality rate in the treatment group. Their study demonstrated that leveraging partnerships and involving citizens, particularly disadvantaged groups (seniors, women, disabled individuals), in the accountability process is a strong facilitating factor of social accountability.

##### Patient charters

A mixed methods study conducted in one Kenyan district found that citizens perceived patients’ rights charters as useful to providing information regarding their local health facility and assisting them with budgeting [27]. The authors studied four facilities: two high ranking and two low ranking. Sixty-six percent of service users reported being aware of the local facility service charter, while the proportion of those who had seen the facility service charter was lowest among one of the two high ranking facilities (50%) and highest among one of the two low ranking facilities (72%). The authors hypothesized that this may be because people reported paying attention to the charter only if they experienced a problem with their service, as is the case with low ranking facilities. Documented barriers to the effectiveness of the charter were that there was a lack of standardization across facilities and that the information included in the charter was fragmented and at times, selective. Health workers also raised fears of reprisal for speaking out after being empowered by the charter. Additionally, in the qualitative component of the study, service users noted that their local culture did not encourage openness and that community issues were often dealt with by community leaders such as tribal chiefs and village elders, thereby weakening the utility of the charter.

#### Unsuccessful interventions

Three (21.4%) of articles reported unsuccessful results from their social accountability approach.

##### Citizen report cards (CRCs)

From a qualitative perspective, Katahoire et al. [22] revealed that although community members, including mothers and caretakers across several Ugandan districts, felt CRCs promoted community dialogue and involvement in monitoring health providers, the type of data included on the CRCs were disputed. For example, responses to the question *why do children sometimes fail to get the medical care they need* were grouped into categories such as “abusive health workers” and “mother sharing doses among children or not giving full doses to children”. These were criticized as not being reflective of all communities. In addition, members with low education levels reported that the CRCs were difficult to understand, and requested more illustrations to complement the statistics. The authors recommended further research and reflection on the type and method of presentation of data on CRCs.

##### Health committees

In an ethnographic study of the Mukono District of Uganda, Golooba-Mutebi et al. [21] reported numerous barriers to effective social accountability based on interviews with health administrative officers and service users. The authors reported that health committees rarely met and were not responsive to citizen complaints about service issues, thereby weakening monitoring of service provision. Participants also expressed that involving powerful third power parties such as established CSOs, NGOs, or local leaders would aid in increasing health provider responsiveness to concerns. Even when citizen concerns reached supervisors and managers, corruption was highlighted as an obstacle to successful reform as ill-behaving health workers were often found to be protected by powerful politicians, thereby undermining the enforceability aspect of social accountability. Similarly, Few et al. [29] reported that in Lusaka, Zambia and Dar es Salaam, Tanzania, there was a low level of awareness of health committees in communities. They also recorded a tendency for community members and committee members to view health committees as bodies designed to service health centres as opposed to the community.

## Discussion

The findings of this systematic review suggest that well-designed and well-implemented social accountability interventions are effective in improving health service quality and outcomes. However, the variability of outcome measures and reporting standards make it difficult to comment on overall effects. In addition, many of the studies, including some of the intervention studies, did not include matched control groups, making it difficult to parse whether reported outcomes are due to the social accountability intervention or due to simply participating in the study. The use of self-reported outcome measures in many studies is also a limitation.

The United Nations’ Sustainable Development Goal 16 (SDG 16) explicitly identifies “effective, accountable, and inclusive institutions at all levels” as essential elements of sustainable development [34]. Our review indicates that successful social accountability interventions involve engaging different sectors and stakeholders, namely community members and health facility staff; ensuring social accountability tools are locally- and contextually- based; integrating data and information collection and tools; fostering trust between citizens and leaders; having clear roles, standards, and responsibilities of those involved in the accountability process; having financial and technical support from experienced groups; and involving citizens and community meaningfully in the process. Limiting factors included lack of motivation from citizens to participate in the implementation of social accountability; fear of reprisal for speaking out; lack of funding and strategic expertise; the amount of time it takes to develop, plan, implement, and evaluate social accountability projects; and lack of government involvement. Only 6 of the 14 social accountability interventions we reviewed were facilitated or supported by a government structure, such as a Ministry of Health or district health management team [14, 22, 24–26, 29]. Furthermore, we observed a lack of timely engagement of citizens in the social accountability process. For example, in the mixed-methods study by Blake et al. [28] using scorecards, citizens were primarily engaged during the scorecard development and assessment phases as opposed to being engaged early on during the process of deciding: what measures should be included in scorecards? How should the scorecard results be presented? The process seemed to be dominated by the researchers, as opposed to making citizen engagement and empowerment central to the process. The approaches used in the cluster-randomized controlled trial by Gullo et al. [33] and the comparative intervention study by Kaseje et al. [30] were consultative and included community and service users to develop the study’s framework and methodology.

Lack of sustainability has been highlighted as an issue of concern for social accountability interventions. The longest duration of follow-up in our study was two years [33]. Rifkin [35] proposes that concepts such as citizen participation and social accountability are better framed as processes, as opposed to interventions, in order to emphasize their long-term nature.

This review provides a comprehensive assessment of facilitating and limiting factors of social accountability interventions in sub-Saharan Africa published over the past 17 years. Strengths include the systematic approach to searching the literature and inclusion of a broad range of study designs. Limitations of the review include restriction of the search to English language studies. There are many French speaking countries in sub-Saharan Africa, where we believe important research on citizen-led accountability has been conducted. Our search was limited to peer-reviewed articles published in scholarly journals, therefore reports from NGOs and CSOs that may have described social accountability initiatives were not considered. Lastly, although every effort was made to be thorough in our search, there is a possibility that we have not included every single social accountability intervention study in our analysis.

Achievement of the post-2015 development goals are contingent upon strong accountability frameworks that involve continuous monitoring and review. Citizen voice has been a key driver in promoting accountability and transparency globally and ensuring health systems respond to the people’s needs.

## Conclusion

Health system fragility and related bottlenecks in Sub-Saharan Africa constrain the achievement of the objectives of global health initiatives and thereby the SDGs. The findings of this review suggest that participatory and deliberative approaches to health policy in sub-Saharan Africa require the engagement of community members and social accountability tools throughout the policymaking cycle. This requires strong institutional support in the form of resources, data, education, and citizen empowerment. Relationship-building between community and leaders in the health sector is vital to fostering a mutual and trustworthy relationship, particularly in contexts of rampant corruption and government mistrust. Social accountability can be mutually beneficial for citizens and health providers, officials, and government. By focusing on citizens as the ultimate beneficiaries of health policies and programs, social accountability provides a mechanism for the empowerment and engagement of citizens with their health system. Future studies implementing social accountability interventions should include sufficiently long periods of follow-up to determine the sustainability of such programs. Stakeholders at national, subnational, regional, and local levels all have a role to play in supporting social accountability initiatives.

## Additional files

Additional file 1: **Table S1.** Characteristics of Included Studies. A summary of the 14 studies included in our review including study design, facilitator(s) and description of accountability initiative, outcome measure(s), key outcomes, and enabling and limiting factors. (DOCX 32 kb)

Additional file 2: **Table S2.** Quality of Included Studies. A summary of our quality assessment of the 14 studies included in our review. (DOCX 19 kb)

## Abbreviations

CHWs: Community health workers
CRC: Citizen report card
CSOs: Civil society organizations
E4A: Evidence for action
GHI: Global health initiatives
HFCs: Health facility committees
MDGs: Millennium development goals
MNH: Maternal and newborn health
NGOs: Non-governmental organizations
SDGs: Sustainable development goals
WHO: World Health Organization
