# Supplementary material for: Improving social accountability processes in the health sector in sub-Saharan Africa: a systematic review
Source: BMC Public Health. 2018 Apr 13;18:497. doi: 10.1186/s12889-018-5407-8 (PMC5899409; doi:10.1186/s12889-018-5407-8)
Supplement: Supplementary file 1 — Table S1. Characteristics of Included Studies. A summary of the 14 studies included in our review including study design, facilitator(s) and description of accountability initiative, outcome measure(s), key outcomes, and enabling and limiting factors. (DOCX 32 kb) [file 12889_2018_5407_MOESM1_ESM.docx]

**Table 1: Characteristics of Included Studies**

| **Author (year)**    **Study Site (Country)**    **Level** | **Study Design Method**  **Facilitator(s) of Accountability Initiative** | **Accountability Initiative (e.g., scorecard, health committee)**    **Outcome Measure(s)** | **Details of Initiative** | **Key outcome(s)**  **Enabling Factors**  **Limiting Factors** |
| --- | --- | --- | --- | --- |
| Atela et al. (2015) [27]    Kenya    District (Kericho) | Mixed  Health facility committee | Local health facility service charter at 4 health facilities    Quantitative component: Household survey, 1024 survey users aged 17+, randomly selected  Qualitative component: 16 focus group discussions analyzed using thematic approach | No intervention implemented; existing intervention described | 66% of respondents had seen the local facility service charter; this proportion was lowest among a low ranking facility (72%) compared to a high ranking facility (50%). 83% of respondents found the charter to be useful or very useful  Strong attention to context, availability of data and information  Perceived lack of responsiveness of health providers, fear of reprisal and punishment |
| Björkman and Svensson (2009) [32]    Uganda    Local/Facility | Quantitative: Community-based, randomized controlled field trial  Local NGOs | Citizen report cards    5,000 household and 50 health provider surveys assessed pre- and post- intervention, over 1 year: Changes in child mortality rates, child weight and treatment practices (immunization rates, waiting time, examination procedures, and absenteeism) after one year | Pre-intervention survey findings compiled into survey, series of meetings held with community, health workers, and health facility members, follow-up every 6 months | After one year:  -36% of treatment facilities had suggestion boxes while no control facilities had them  -70% of treatment facilities also posted information on free services and patients’ rights compared to only 4 out of 25 control clinics  -Waiting time - 131 min in control facility and 119 min in treatment facility  -Absenteeism: 13 percentage points lower in treatment facility  -33% reduction in under-5 mortality rate  Leveraging partnerships, building coalitions, timely interventions, strong attention to context |
| Blake et al. (2016) [28]    Ghana    District | Mixed  Evidence 4 Action (E4A) Program | Scorecards    Quantitative component: Pre- and post- intervention facility assessment questionnaires after one year of 37 health facilities, completed by multiple health and non-health stakeholders including service users (*N* not reported)  Qualitative component: Impact of changes in policy, attitudes, and/or practices analyzed using content analysis | Scorecards developed based on questionnaire result; stakeholder meetings facilitated at district/municipal, health facility, and community level; nine member assessment team composed of 4 administrative health official members, political member, 1 community-based organization member, and 3 council leaders; results from meetings made public | After one year:  -41% increase in essential drugs rating, 22% increase in infrastructure rating, 47% increase in accessibility and access to information rating, 14% increase in water, sanitation, and hygiene rating, 18% increase in essential equipment rating  -Overall 24% increase in facility infrastructure and assessment rating and 14% increase in client perspectives assessment rating  Strong attention to context, leveraging partnerships/building coalitions, clear roles/responsibilities/standards, timely interventions  Lack of financial and technical inputs, differing expectations between community and leaders, weak leadership at local and district levels |
| Few, Harpham, and Atkinson (2003) [29]    Zambia and Tanzania    Municipal (Lusaka and Dar es Salaam) | Mixed  Government health sector reforms | Health Facility Committees    Qualitative analysis involved desk review of documentation, health facility staff interviews and focus groups, exit interviews with health service users and a community household survey. Midterm evaluations conducted  Lusaka: Baseline and Follow-up  Dar es Salaam: No baseline | Part of a larger study. Social accountability component included supporting the formation of community health committees which worked with health centre staff to identify and manage health system problems | Quantitative improvements in health service quality or outcomes not reported. Health staff reported increased tendency for users to voice complaints and better awareness of their rights  Health committee role in monitoring, planning and management strengthened throughout project; clear roles, responsibilities, and standards; leveraging partnerships  Low level of awareness in communities of the committees, tendency for community members and committee members to view health committee as body designed to service health centre, not the community. Lack of financial and technical resources; role conflict; citizen disengagement and lack of knowledge |
| Golooba-Mutebi (2005) [21]    Uganda    District (Mukono) | Qualitative  Local Council | Health Committees    3 month study involving participation observation at health facilities and events, interviews with patients and health workers, document analysis | Part of a larger, 9-month study of the decentralization of Uganda’s health system. No intervention implemented; existing intervention described. Three categories of health committee formed: district health and environment committees, sub-county health committees, and health-unit management committees | Health committees did not met regularly, complaints by service users rarely received a response  Leveraging partnerships; clear roles, responsibilities, and standards; strong attention to context  Corruption in government, weak state; citizen disengagement and lack of knowledge; lack of funding support for health committees |
| Gullo et al. (2017) [33]  Malawi  District (Ntcheu) | Quantitative: Cluster-randomized controlled trial  CARE Malawi | Scorecards  Quantitative component: Pre- and post- intervention assessments that evaluated modern contraceptive use, antenatal and postnatal care service utilization, perceived service quality, and service satisfaction. Controlled for demographic factors (e.g., religion, ethnicity)  Baseline: 1,301 women who recently gave birth  Evaluation: 1,300 women who recently gave birth | CARE’s citizen scorecard assessed in 20 health facilities, health facilities matched and 1 of the pair was randomly assigned to receive intervention. 5 phase project: planning and preparation (training facilitators, securing buy-in of stakeholders such as govt), focus group with community members to develop scorecard indicators, indicators further developed with health providers, interface meeting to bring together community, health providers, and officials, and finally, action plan implementation, monitoring, and evaluation. Cycle repeated every 6 months | After two years:  -In the intervention group, CHW visits to pregnant women increased by 20% and by 6% in the postnatal period, compared to control group  -Women’s satisfaction with health services increased significantly  -37% increase in rating on relationship between health provider and community  -22% increase in ratings on availability and accessibility of reproductive and maternal health information  -Other significant increases in ratings of commitment of service providers (26%), level of youth and male involvement (23 and 33%, respectively), and availability of referral transportation (21%)  Leveraging partnerships; clear roles, responsibilities, and standards; strong attention to context; availability of funding and expertise |
| Hoope-Bender et al. (2016) [14]    Nigeria, Sierra Leone    State (Bauchi, Nigeria), District | Qualitative  Evidence 4 Action and Ministry of Health | Scorecards    Not directly reported. Measure reported in Nigeria was change in government health budget | Nigeria: State accountability mechanism initiated in 2014, wide range of stakeholders including CSOs involved in translating MNH evidence into scorecards, follow-up advocacy activities involving citizens and youth targeted towards MoH officials, media also used  Sierra Leone: The Budget Advocacy Group, a coalition of CSOs, developed scorecards in 2012 during election period, used by citizens to question electoral candidates on commitments, TV and radio also used | Nigeria: Bauchi State Government’s budget increased from 8% (2014) to 15% (2016); Sierra Leone: Pledges signed by 68 parliamentarians and 5 out of 6 political parties signed a “Health Manifesto”  Timely interventions; leveraging partnerships/building coalitions; availability of data and information  Lack of financial and technical resources |
| Kaseje et al. (2010) [30]    Kenya    District (Nyando, Rachuonyo, Siaya, Kisimu, Bondo, Suba) | Mixed  Great Lakes University of Kisumu | Multiple used: Health committees, suggestion boxes, and so on    Quantitative component: Controlled comparative intervention study -  Two year pre- and post- intervention assessment in 6 districts of the following parameters: Immunization coverage, health facility delivery, ITN availability, ANC visits, household water treatment (%), food availability, latrine presence and FP usage  I: *n* = 17,668  C: *n* = 1,817  Qualitative component: Two key informants and 1 focus group discussion with CHWs analyzed via thematic analysis | Consultative workshops held with service users, policy makers, managers, government officials, and communities to develop conceptual framework and study methodology. Three-day training workshops held 3 times during intervention period to introduce and explain the intervention. Dialogue sessions held monthly at community level and quarterly at health facility and district management level. | Immunization coverage was significantly higher at intervention sites (91%) vs. control sites (66%); birth delivery rates at health facility remained low  Leveraging partnerships; availability of data and information; clear roles, responsibilities, and standards  Lack of sufficient data collection and management tools, non-contextual targeted initiatives |
| Katahoire et al. (2015) [22]    Uganda    District (Bukomansibi, Masaka, Buikwe, Mukono, Wakiso) | Qualitative  The Community and District Empowerment for Scale-Up project and implementing partners including district health management | Citizen Report Cards    38 semi-structured interviews with District Health Team members and implementing partners across 5 districts, observationational methods, and document review conducted over 2 years. Analyzed using thematic approach | First 2 years were proof-of-concept. CRCs developed using Lot Quality Assurance Sampling surveys and qualitative surveys, CRCs revised based on feedback. Two-day community dialogues involving 70-100 parents and children, community leaders, and health workers were conducted, involved interface meetings, signing commitment contracts, and electing a monitoring committee. Participants encouraged to use U-Report, an anonymous SMS monitoring tool, to provide feedback | Low response rate of U-Report community feedback mechanism. Community dialogues using CRCs increased community knowledge of available and quality of services and involved citizens in decision-making processes    Leveraging partnerships; availability of data and information; clear roles, responsibilities, and standards  Lack of financial and technical resources, unequal power relations, poor institutionalization of social accountability mechanisms, contextual considerations (e.g., illiterate mothers may not be familiar with SMS technology) |
| Lodenstein et al. (2017) [23]    Benin, Guinea, and Democratic Republic of Congo (DRC)    Facility/Local | Qualitative  Royal Tropical Institute, Netherlands | Health Facility Committees    Examination of 11 HFCs across 3 countries from Jan to April 2014 via document analysis, 95 interviews, and 22 focus group discussions with citizens, health workers, committee members, and local authorities. Sources triangulated | Interviews and focus group discussions focused on three target areas: HFC and health facility characteristics, examples of social accountability practices, and participants’ perceptions of the effectiveness of social accountability relations and practices | HFCs initiate information and data collection, provide a forum for dialogue, ensure consequences and follow-up of complaints, and provide feedback to the community  Leveraging partnerships/building coalitions; strong attention to context; availability of data and information  Poor institutionalization of social accountability mechanisms; lack of financial and technical resources; legitimacy of NGOs and community groups |
| Mafuta et al. (2015) [24]    DRC    Health Zone (Muanda and Bolenge) | Qualitative  Health Centre/Government | Varied, mainly HFCs    Semi-structured interviews conducted between Sept and Oct 2014 with 25 women, 5 men, 5 health providers, 2 health zone officers, and 11 community stakeholders on women’s expectations, health services, voicing concerns, and monitoring and enforcing health provider performance. Analyzed via thematic content analysis | No intervention implemented; existing intervention described | Women rarely voiced concerns about health services, citing fear of reprisal, lack of knowledge of procedures and rights, and power imbalances. CHWs, HFCs, and community-based organizations identified as oversight mechanisms. HFCs not well known  Leveraging partnerships/building coalitions; strong attention to context  Fear of reprimand or punishment; perceived lack of health provider responsiveness; citizen disengagement and lack of knowledge; unequal power relations; poor institutionalization of social accountability mechanisms |
| Mafuta et al. (2016) [25]    DRC    Health Zone (Muanda and Bolenge) | Qualitative  Health Centre/Government | Varied, mainly HFCs  Semi-structured interviews with 5 CHWs and health committee members, 13 community groups, 3 community leaders, 2 health partners, 3 health providers, 5 health zone management officers, and 2 local public administration officers; and document analysis from May to June 2013. Analyzed via thematic analysis | No intervention implemented; existing intervention described | Facilitators of SA: community associations and groups, experiences in social mobilization and networking, cultural diversity and marginalized population, women’s status and participation in community groups’ activities, existing media and access to information, supportive regulatory environment, resources, and negotiation ability. Limiting factors: Lack of networks, insufficient capacity for community mobilization, poor socioeconomic conditions, lack of media coverage in underserved areas, and poor negotiation ability  Strong attention to context; availability of data and information  Unequal power relations; lack of financial and technical resources; lack of political capital |
| Mafuta et al. (2017) [26]    DRC    Health Zone (Muanda and Bolenge) | Qualitative  Health Centre/Government | HFCs    *N* = 121 Focus groups involving 121 individuals from the health sector and local authorities, CHWs and health committee members, men’s and women’s group representatives, and health partners. Analyzed via inductive content analysis | Uses the Dialogue Model, which emphasizes a joint learning process among stakeholders and a participatory research action approach. Four phases involved: exploration and preparation based on surveys and interviews, consultation and prioritization which involved focus group discussions, integration and prioritization which involved a joint dialogue meeting, programming, and implementation | Six intervention components derived: Use CHWs and HFCs to address community concerns, build community capacity in terms of knowledge and information, engage community leaders, improve the attitude of health providers towards voice, improve health facility management attitude towards community participation, and use other existing interventions  Leveraging partnerships; strong attention to context; timely interventions  Lack of financial and technical inputs; poor institutionalization of social accountability mechanisms; lack of female involvement and empowerment; perceived lack of health provider responsiveness |
| Uzochukwu, Akpala, and Onwujekwe (2004) [31]    Nigeria    District and Village (Oji River local government area) | Mixed  Nigerian Government | HFCs    Quantitative component: Self-administered questionnaire from 20 facility heads assessing knowledge of village health committees (VHCs) and their composition and functionality  Qualitative component: Focus group discussions with 9-10 members of the district and VHCs. Analyzed via thematic analysis | No intervention implemented; existing intervention described | Overall positive perceptions of HFCs: For example, 89.3% and 100% of village- and district- level committee members reported changes in drug provision, 100% believe in participating in community mobilization  Overwhelmingly positive response to health committees: health workers perceived positively (e.g. 95% of health workers were members of the committees, 95% had female members, 85% were satisfied with the functionality  Clear roles, responsibilities and standards; timely interventions  Top-down hierarchical decision-making; unequal power relations; poor conceptualization of community participation; lack of financial and technical resources; citizen disengagement and lack of knowledge |

SA: Social Accountability

HFC: Health Facility Committee

I: Intervention

C: Control

VHC: Village Health Committee
