# Supplementary material for: Improving social accountability processes in the health sector in sub-Saharan Africa: a systematic review
Source: BMC Public Health. 2018 Apr 13;18:497. doi: 10.1186/s12889-018-5407-8 (PMC5899409; doi:10.1186/s12889-018-5407-8)
Supplement: Supplementary file 2 — Table S2. Quality of Included Studies. A summary of our quality assessment of the 14 studies included in our review. (DOCX 19 kb) [file 12889_2018_5407_MOESM2_ESM.docx]

**Table 2: Quality of Included Studies***

| **Ref. #** | **Author (Year), Country** | **Focus on SA** | **Operationalized concept of SA** | **Baseline assessment?** | **Intervention study: control group?** | **Qualitative study: method of analysis described?** | **Quantitative study: Objective outcome measure(s)?** | **SA approach specified?** | **Population of interest described?** | **Total Score** |
| --- | --- | --- | --- | --- | --- | --- | --- | --- | --- | --- |
| 27 | Atela et al. (2015), Kenya | 1 | 1 | 0 | n/a | 1 | 1 | 1 | 1 | 6/8 |
| 32 | Bjorkman and Svensson (2009), Uganda | 1 | 0 | 1 | 0 | n/a | 1 | 1 | 1 | 5/7 |
| 28 | Blake et al. (2016), Ghana | 1 | 0 | 1 | 0 | 1 | 1 | 1 | 1 | 6/8 |
| 29 | Few et al. (2003), Zambia and Tanzania | 1 | 0 | 0.5 | n/a | 0 | n/a | 1 | 1 | 3.5/6 |
| 21 | Golooba-Mutebi (2005), Uganda | 1 | 0 | 0 | n/a | 1 | n/a | 1 | 1 | 4/6 |
| 33 | Gullo et al. (2017), Malawi | 1 | 1 | 1 | 1 | n/a | 1 | 1 | 1 | 7/7 |
| 14 | Hoope-Bender et al. (2016), Nigeria and Sierra Leone | 1 | 0 | 0 | n/a | 0 | n/a | 1 | 0 | 2/6 |
| 30 | Kaseje et al. (2010), Kenya | 0 | 0 | 1 | 1 | 1 | 1 | 1 | 1 | 6/8 |
| 22 | Katahoire et al. (2015), Uganda | 0 | 0 | 1 | n/a | 1 | n/a | 1 | 1 | 4/6 |
| 23 | Lodenstein et al. (2017), Benin, Guinea, and DRC | 1 | 1 | 0 | n/a | 1 | n/a | 1 | 1 | 5/6 |
| 24 | Mafuta et al. (2015), DRC | 1 | 1 | n/a | n/a | 1 | n/a | 1 | 1 | 5/5 |
| 25 | Mafuta et al. (2016), DRC | 1 | 1 | n/a | n/a | 1 | n/a | 1 | 1 | 5/5 |
| 26 | Mafuta et al. (2017), DRC | 1 | 1 | n/a | n/a | 1 | n/a | 1 | 1 | 5/5 |
| 31 | Uzochukwu, Akpala, and Onwujekwe (2004), Nigeria | 0 | 0 | n/a | n/a | 1 | 1 | 1 | 1 | 4/6 |

*Score of 0 = not present, 1 = present

SA: social accountability
